# RAMAS-Net: a module-optimized convolutional network model for aortic valve stenosis recognition in echocardiography

**DOI:** 10.3389/fmed.2025.1587307

**Published:** 2025-04-28

**Authors:** Yejia Gan, Wanzhong Huang, Yan Deng, Xiaoying Xie, Yuanyuan Gu, Yaozhuang Zhou, Qian Zhang, Maosheng Zhang, Yangchun Liu

**Affiliations:** ^1^Department of Information and Management, Guangxi Medical University, Nanning, China; ^2^Department of Cardiology, Jiangbin Hospital of Guangxi Zhuang Autonomous Region, Nanning, China; ^3^Department of Ultrasound, The First Affiliated Hospital of Guangxi Medical University, Nanning, China; ^4^Department of Cardiovascular Medicine, The Third Affiliated Hospital of Sun Yat-sen University, Guangzhou, China; ^5^Cardiothoracic Surgery Intensive Care Unit, The First Affiliated Hospital of Guangxi Medical University, Nanning, China

**Keywords:** deep learning, aortic valve stenosis, cardiovascular disease, echocardiography, transthoracic-echocardiography, artificial intelligence

## Abstract

**Introduction:**

Aortic stenosis (AS) is a valvular heart disease that obstructs normal blood flow from the left ventricle to the aorta due to pathological changes in the valve, leading to impaired cardiac function. Echocardiography is a key diagnostic tool for AS; however, its accuracy is influenced by inter-observer variability, operator experience, and image quality, which can result in misdiagnosis. Therefore, alternative methods are needed to assist healthcare professionals in achieving more accurate diagnoses.

**Methods:**

We proposed a deep learning model, RSMAS-Net, for the automated identification and diagnosis of AS using echocardiography. The model enhanced the ResNet50 backbone by replacing Stage 4 with Spatial and Channel Reconstruction Convolution (SCConv) and Multi-Dconv Head Transposed Attention (MDTA) modules, aiming to reduce redundant computations and improve feature extraction capabilities.

**Results:**

The proposed method was evaluated on the TMED-2 echocardiography dataset, achieving an accuracy of 94.67%, an *F*_1_-score of 94.37%, and an AUC of 0.95 for AS identification. Additionally, the model achieved an AUC of 0.93 for AS severity classification on TMED-2. RSMAS-Net outperformed multiple baseline models in recall, precision, parameter efficiency, and inference time. It also achieved an AUC of 0.91 on the TMED-1 dataset.

**Conclusion:**

RSMAS-Net effectively diagnoses and classifies the severity of AS in echocardiographic images. The integration of SCConv and MDTA modules enhances diagnostic accuracy while reducing model complexity compared to the original ResNet50 architecture. These results highlight the potential of RSMAS-Net in improving AS assessment and supporting clinical decision-making.

## 1 Introduction

Aortic stenosis (AS) is a frequently occurring valvular heart disease that is mainly due to the narrowing or hardening of the aortic valve (AV), which impacts the normal blood flow from the left ventricle to the aorta (1, 2). This condition can increase the cardiac burden and, in severe cases, lead to heart failure. Valve stenosis is usually caused by aging, calcium deposition, or congenital diseases (3). In the early stages of aortic stenosis, patients may have no noticeable symptoms or very mild symptoms that are often overlooked. As the condition progresses and stenosis worsens, it may lead to exertional dyspnea, chest pain, and syncope, all resulting from inadequate cardiac perfusion. In advanced stages, left ventricular dilation, decreased wall elasticity, and impaired systolic function can lead to heart pump failure and possible blood regurgitation (4). With an aging population, aortic stenosis is becoming an increasingly common issue. Clinically, severe AS is a potentially fatal condition, with untreated moderate to severe AS patients having five-year mortality rates of 56 and 67%, respectively (5). Therefore, creating an easily accessible screening method is essential for prompt diagnosis and immediate intervention.

**Figure 1:**
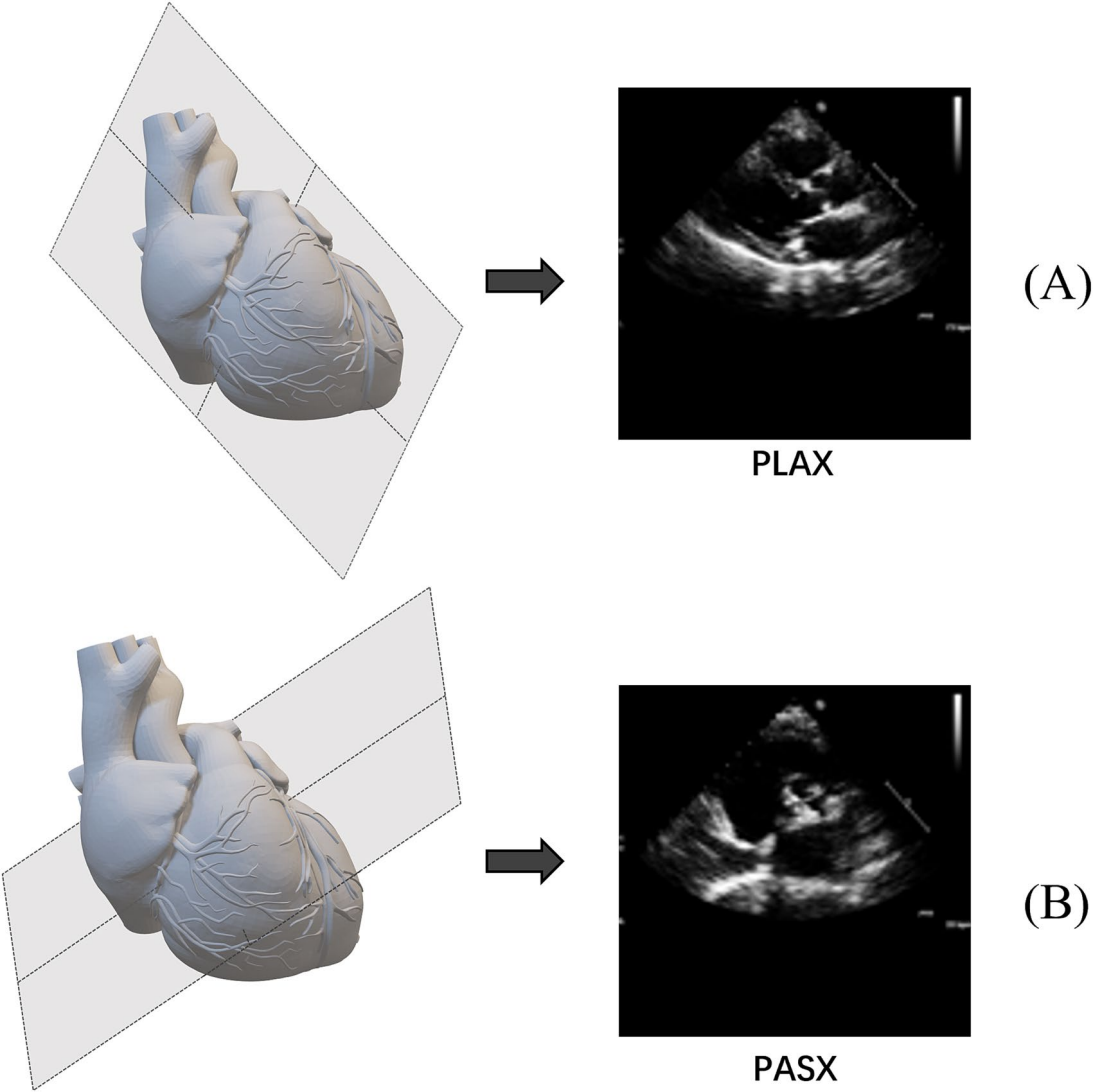
Examples of common echocardiographic diagnostic views. **(A)** Parasternal long-axis (PLAX). **(B)** Parasternal short-axis (PSAX).

Transthoracic echocardiography (TTE) is one of the key tools for diagnosing aortic stenosis. It is a widely used cardiac imaging technique that assesses the heart’s structure and function (6) using an ultrasound probe on the chest wall. Echocardiography encompasses several views such as parasternal long-axis (PLAX), parasternal short-axis (PSAX), apical two-chamber (A2C), and apical four-chamber (A4C), all of which are used for diagnosing AS. PLAX images the heart along its long axis, usually taken from the third or fourth intercostal space at the left sternal border, as shown in Figure 1A. PSAX images the heart along its short axis, also from the left sternal border but with the probe oriented differently, as shown in Figure 1B. These views provide detailed information to help doctors assess the valve’s structure, function, and the severity of the pathology. Specifically, the information includes valve morphology and closure, valve orifice area, average blood flow velocity and pressure gradient, as well as the extent of valve calcification (7). Both A2C and A4C image the heart from the apex, but with different probe orientations and angles. These views allow the indirect evaluation of aortic stenosis by observing the degree of left ventricular hypertrophy, the extent of left ventricular outflow tract narrowing, and the aortic root dilation. Doctors evaluate these parameters to diagnose AS. However, factors such as the operator’s image acquisition skills, Doppler usage techniques, and echocardiogram interpretation abilities can lead to poor reproducibility, misdiagnosis, and increased inter-observer variability. In routine clinical assessments of AS severity, 20–30% of cases may yield conflicting results regarding stenosis severity (8). Therefore, there is a need for alternative methods to assist clinicians in making accurate diagnoses.

Deep learning’s powerful feature learning and pattern recognition capabilities enable it to address the characteristics of echocardiography and the aforementioned issues, achieving excellent automatic identification and analysis. This provides an efficient and accurate tool for clinical use, making automated assisted diagnosis feasible.

In the study by Ghorbani et al. (9), deep learning was not only used to identify local structures of the heart and assess cardiac function but also to predict detailed cardiac structures, evaluate heart function, and predict physiological characteristics such as age and gender. Liu et al. (10) developed a deep learning framework called AIEchoDx, specifically for diagnosing cardiovascular diseases and locating lesions in echocardiography. It can distinguish four common cardiovascular diseases from echocardiograms and accurately identify key lesion areas for each disease, demonstrating the efficiency of deep learning in disease localization and heterogeneity typing. The review study by Hassan and Obied (11) discussed in detail the application of different deep learning techniques in cardiac disease classification. By analyzing existing research, the authors emphasized the role of deep learning in improving the accuracy of echocardiographic analysis.

These studies show considerable advancements in applying deep learning to ultrasound image recognition, particularly in processing and analyzing echocardiographic data, which significantly improves diagnostic accuracy and efficiency. This includes the identification of aortic stenosis. For example, Holste et al. (12) proposed a 3D convolutional neural network for identifying severe AS using PLAX-view echocardiography videos. The model was pretrained via self-supervised learning, fine-tuned through ensemble learning across datasets, and finally trained with supervised learning. Hatfaludi et al. (13) developed a deep learning model based on Faster R-CNN with VGG and ResNet backbones to detect and classify aortic valve states from PLAX images, using a temporal model to aggregate frame-level features. Ahmadi et al. (14) introduced a spatiotemporal Transformer-based architecture that integrates anatomical and motion features from 2D echocardiographic data, achieving high accuracy in classifying AS severity on both public and private datasets. Avola et al. (15) introduced a multi-view multi-scale feature extractor and transformer encoder (MV-MS-FETE) designed to predict valve stenosis using parasternal long-axis and short-axis views. Dual feature extractors generate multi-scale maps, which are then sequentially combined and passed to a patch embedding module to create latent representations of the ultrasound images. These representations are then fed into a transformer encoder to identify whether aortic valve stenosis is present.

Based on the aforementioned conceptual background and literature review, the automated diagnosis of AS still faces several critical challenges. First, existing models typically have large parameter sizes, making them difficult to deploy in clinical settings that require lightweight architectures and real-time responsiveness. Second, most current approaches rely on a single echocardiographic view (e.g., PLAX or PSAX), failing to fully leverage multi-view information. Third, classification performance—including accuracy, recall, and other key metrics—remains suboptimal and requires further improvement. Given the often asymptomatic nature of early-stage AS and the high rate of clinical misdiagnosis, developing an efficient, accurate, and clinically applicable screening tool is essential to improve early detection and facilitate timely intervention.

In practical clinical applications, improving the diagnostic accuracy and interpretive consistency of echocardiographic analysis can reduce errors caused by subjective human judgment and enhance reproducibility. Additionally, compared to high-cost imaging modalities such as magnetic resonance imaging (MRI), ultrasound offers greater affordability and convenience, making it better suited for large-scale screening and preliminary evaluation. Therefore, developing cost-effective diagnostic models based on ultrasound images can reduce dependence on expensive equipment and improve the efficiency of medical resource utilization. Furthermore, by enabling automated analysis and interpretation of echocardiographic images, deep learning models have the potential to promote intelligent AS diagnostic workflows, alleviate the workload on clinicians, and enhance overall healthcare efficiency.

To address these challenges, this study proposes a structurally optimized and lightweight deep learning model for binary classification of AS. The model is built upon the ResNet50 backbone and integrates Spatial and Channel Reconstruction Convolution (SCConv) and Multi-Dconv Head Transposed Attention (MDTA) modules to enhance feature extraction and classification performance. We name this model RSMAS-Net, which is designed to improve the accuracy and efficiency of AS recognition in echocardiographic images, providing a reliable and clinically practical tool for intelligent diagnostic support.

In summary, this paper’s contributions include the following:

1. Proposal of RSMAS-Net: This study introduces RSMAS-Net, a module-optimized convolutional neural network specifically designed for identifying AS in echocardiography. By integrating SCConv and MDTA attention modules into the ResNet50 backbone, the proposed network effectively reduces redundant computations, enhances representative feature learning, and improves the accuracy and efficiency of AS recognition tasks.
2. Superior performance and benchmark establishment: RSMAS-Net achieves higher classification accuracy than several widely used CNN models (ResNet50, EfficientNet, MobileNet, SqueezeNet, and VGG16) on the TMED-2 dataset, while requiring fewer parameters and offering faster inference speed. Moreover, the proposed model demonstrates robust generalization performance on the TMED-1 validation dataset. These results establish new benchmark references for future research in echocardiographic AS classification.
3. Advancement of AI-assisted echocardiographic diagnostic workflows: This study presents an efficient and accurate deep learning model tailored for automated AS diagnosis, promoting the practical integration of deep learning techniques into echocardiographic analysis. The proposed model can serve as a key component of intelligent diagnostic systems, providing technical support for the development of AI-driven echocardiographic workflows.

## 2 Materials and methods

This section focuses on the approach we adopted in our improved deep learning model, RSMAS-Net, for aortic stenosis identification in this research. The proposed model is named RSMAS-Net, where R represents the backbone network (ResNet50), S stands for the introduced SCConv module, M refers to the integrated MDTA attention module, and AS denotes the target clinical condition, aortic valve stenosis. The full name of the model is ResNet50 with SCConv and MDTA Attention for Aortic Stenosis Classification Network.

**Figure 2:**
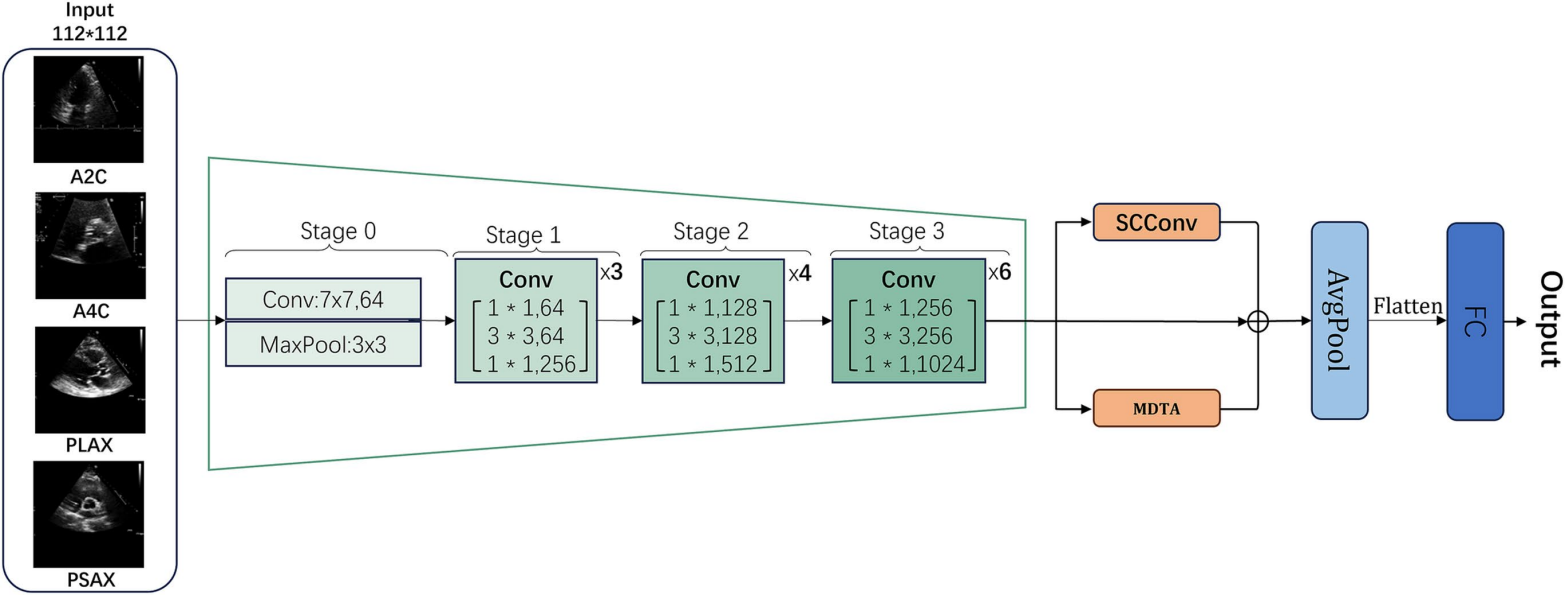
General workflow structure of RSMAS-Net model.

RSMAS-Net is built upon the ResNet50 framework and has been modified to address the classification and diagnostic characteristics of echocardiography. In the original ResNet50 structure, we replaced the convolutional set in the original fourth stage (Stage 4) with a combination of SCConv and MDTA modules, further boosting the model’s capacity to understand and represent echocardiographic characteristics, as shown in Figure 2. The inclusion of these two modules aims to utilize the SCConv module to lessen redundant computations and promote the acquisition of representative features, and the MDTA module’s multidimensional attention mechanism to more effectively identify and diagnose aortic stenosis in echocardiography. Through these optimizations, the model demonstrates higher accuracy in identifying variant cardiac pathological conditions, such as aortic stenosis.

Building upon the ResNet50 framework, RSMAS-Net introduces targeted innovations to overcome the limitations of conventional architectures when applied to echocardiographic image analysis. The original Stage 4 of ResNet50 consists of multiple residual blocks containing standard convolutional layers and skip connections. However, traditional ResNet50 may face limitations in handling echocardiographic images, including information redundancy, insufficient local feature representation, and limited capability in modeling long-range dependencies. To overcome these issues, we replaced and enhanced this stage at the module level.

Specifically, we replaced the convolutional layers in Stage 4 with a combination of SCConv (Spatial-Channel Convolution) and MDTA (Multi-Dimensional Transformer Attention) modules to more effectively extract and represent critical pathological features in echocardiographic images:

SCConv Module (Section 2.2): This module integrates spatial and channel information, reducing redundant computations while enhancing feature representation. By focusing on key anatomical structures in echocardiographic images, SCConv minimizes background noise interference and increases sensitivity to subtle pathological changes, all without significantly increasing computational cost compared to standard convolution.

MDTA Module (Section 2.3): The MDTA module employs a multi-dimensional self-attention mechanism that captures multi-scale feature information, enhancing the model’s ability to fuse local and global information. Given the dynamic nature and spatial dependencies of echocardiography, MDTA strengthens cross-region associations, improving pathological pattern recognition in ultrasound images.

Additionally, we incorporated a global average pooling (AvgPool) layer at the final stage to reduce model parameters, mitigate overfitting, and streamline computation, followed by a fully connected (FC) layer to output classification results.

This structural optimization improves AS detection accuracy while maintaining an optimal balance between generalization ability and computational efficiency. By integrating these enhancements, the model achieves more accurate recognition of AS lesions in echocardiographic images, demonstrating robustness to pathological variations and greater clinical applicability in computer-aided diagnosis.

### 2.1 RSMAS-Net backbone network ResNet50

ResNet (Residual Network) is a deep convolutional neural network proposed by He et al. (16) to address the training difficulties of deep networks. By introducing residual blocks, it allows inputs to be directly passed to subsequent layers through skip connections. This paper uses ResNet50 as the main framework of the model. The ResNet series includes various structures depending on the network depth, and ResNet50 is one of them, comprising 50 neural network layers. One convolution operation is contained in the first group of convolutions, also known as Stage 0. The second through fifth convolutional groups comprise several identical residual units. In the code implementation, these are usually referred to as Stage 1, Stage 2, Stage 3, and Stage 4, respectively. Stages 1–3 contain 3, 4, and 6 Bottleneck modules, respectively. The TMED-2 public dataset used in this study is suitable for the classification training of small sample image dataset models. Therefore, while maintaining a moderate overall parameter count, the model possesses good network performance and excellent feature extraction capabilities. Considering these factors, this paper selects the ResNet50 network as the backbone for further improvement.

### 2.2 SCConv module

**Figure 3:**
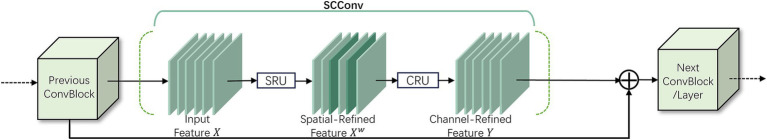
Overall framework of the SCConv module.

Spatial and Channel Reconstruction Convolution (SCConv) is an optimized component created by Li et al. (17) to mitigate feature redundancy within convolutional neural networks. In deep learning networks, there is notable redundancy present in model parameters as well as in the spatial and channel aspects of feature maps. SCConv minimizes unnecessary computations and improves the learning of key features by tackling both spatial and channel redundancies, thus enhancing computational efficiency and overall performance. Figure 3 shows the framework of SCConv.

SCConv is created to function as a plug-and-play component, meaning it is readily integrable within established convolutional neural network frameworks, replacing traditional convolutions without significant modifications. Its primary components are the Spatial Reconstruction Unit (SRU) and the Channel Reconstruction Unit (CRU). The specific workflow is as follows:

First, the feature map Χ processed by the previous convolutional block is received. It is input into the Spatial Reconstruction Unit (SRU), where a series of operations, including group normalization and thresholding, separate and reconstruct features to reduce spatial redundancy, resulting in spatially refined features ${Χ}^{w}$ . The Channel Reconstruction Unit (CRU) receives ${Χ}^{w}$ output from the SRU and reduces channel redundancy through segmentation, transformation, and fusion strategies, generating channel-refined features **Y**. The channel-refined features **Y** output by the CRU are processed through convolution, then added to the previous features, and finally passed to the next layer. This design aims to reduce redundancy in feature extraction, enhancing the efficiency and effectiveness of convolutional neural networks for feature processing.

#### 2.2.1 Spatial Reconstruction Unit

**Figure 4:**
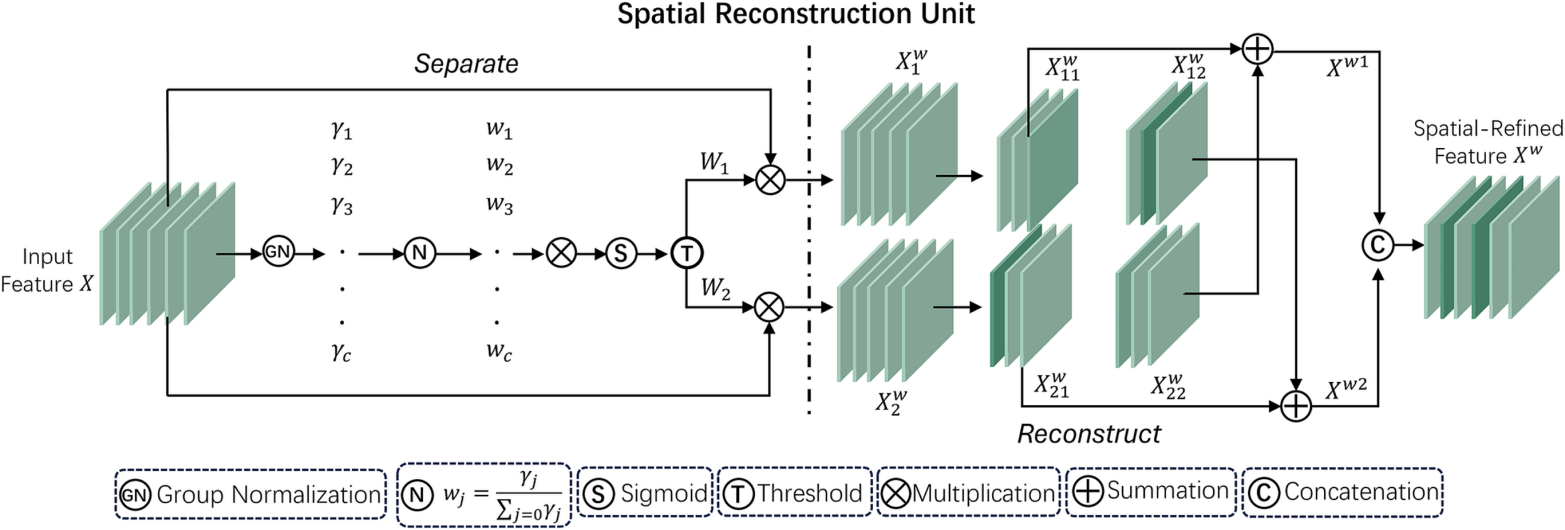
Workflow structure of the SRU unit within the SCConv module.

This unit uses a “separation-reconstruction” method to manage spatial redundancy. It separates less informative features from more valuable ones, refining the extracted spatial features to enhance overall feature representation. The SRU structure is depicted in Figure 4.

In this process, the scaling factors of the Group Normalization (GN) (18) layer are used to evaluate the information content of various feature maps. Specifically, for the input intermediate feature map $Χ\in {\mathbb{R}}^{N\times C\times H\times W}$ , where *N*, *C*, *H*, *W* represent batch, channel, height, and width dimensions, respectively, the initial step involves normalizing the input features Χ by removing their mean *μ* and scaling by their standard deviation *σ*:


$${Χ}_{\mathrm{out}}=GN\left(X\right)=\gamma \frac{Χ-\mu }{\sqrt{\sigma^{2}+\varepsilon }}+\beta$$

Here, the parameters *μ* and *σ* denote the feature map’s mean and standard deviation, *ε* is added for numerical stability, and *γ* and *β* adjustable parameters in the GN layer.

The vector γ (in ${\mathbb{R}}^{c}$ ) is used to evaluate the spatial pixel variability for each channel and batch. Higher variability indicates richer spatial information, typically resulting in larger values. Then, the normalized weights of the feature map $W_{\gamma }\in {\mathbb{R}}^{C}$ are calculated using the following formula:


$$W_{\gamma }=\left\{\omega_{i}\right\}=\frac{\gamma_{i}}{\sum_{j=1^{\gamma j}}^{C}},i,j=1,2\cdots ,C$$

These weights are then converted to the (0, 1) range using a sigmoid function and selectively gated through a threshold. Weights exceeding the threshold are set to 1 (representing informative features, denoted as $W_{1}$ ), while the rest are set to 0 (representing less informative features, denoted as $W_{2}$ ); In the experiments, the threshold is established at 0.5. This way, the input features *Χ* are effectively separated based on their information content.


$$W=\mathrm{Gate}\left(\mathrm{Sigmoid}\left(W_{\gamma }\left(GN\left(X\right)\right)\right)\right)$$

Furthermore, to address spatial redundancy, a “reconstruction” operation is implemented to enhance feature representation and conserve space by overlaying informative features with less informative ones. A cross-reconstruction strategy is employed instead of direct addition to effectively integrate the two types of information. The reconstructed features ${Χ}_{\omega 1}$ and ${Χ}_{\omega 2}$ are then concatenated to obtain the optimized spatial feature map ${Χ}_{\omega }$ . The entire reconstruction process can be described as follows:


$$\left\{ \begin{array}{c} {Χ}_{1}^{w}=W_{1}⊗Χ,\\ {Χ}_{2}^{w}=W_{2}⊗Χ,\\ {Χ}_{11}^{w}⊕{Χ}_{22}^{w}={Χ}^{w1},\\ {Χ}_{21}^{w}⊕{Χ}_{12}^{w}={Χ}^{w2},\\ {Χ}^{w1}\cup {Χ}^{w2}={Χ}^{w} \end{array}\right.$$

where ⊗ signifies element-wise multiplication, ⊕ signifies element-wise summation, and ∪ signifies the Concat concatenation module.

#### 2.2.2 Channel Reconstruction Unit

**Figure 5:**
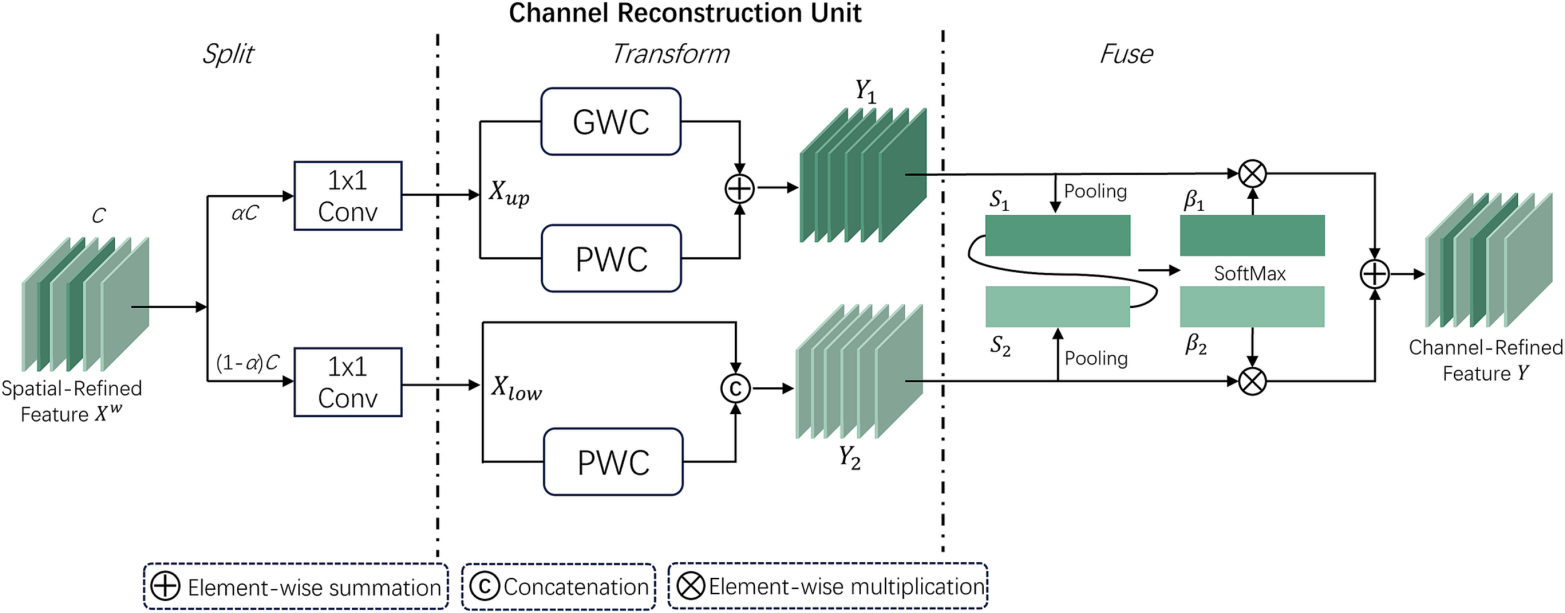
Workflow structure of the CRU unit within the SCConv module.

Although the spatially optimized feature maps are improved in the spatial dimension, they may still have redundancy in the channel dimension. Therefore, the CRU unit uses a “split-transform-fuse” strategy to further address channel redundancy. The CRU unit involves three operations: Split, Transform, and Fuse. These operations maintain effective information flow and reduce computational costs. The structure is depicted in Figure 5.

In the split stage, the channels of the feature map ${Χ}^{w}$ are first divided into two groups, *αC* and (1 − *α*)*C*, and then compressed using 1 × 1 convolution. A compression ratio *r* is introduced to control the feature channels of the CRU, optimizing computational efficiency and balancing computational costs. The spatially refined features ${Χ}^{w}$ are then divided into ${Χ}_{\mathrm{up}}$ and ${Χ}_{\mathrm{low}}$ .

In the transform stage, ${Χ}_{\mathrm{up}}$ is sent to the upper transform stage, acting as a “rich feature extractor.” Efficient convolution operations GWC (Group-Wise Convolution) and PWC (Point-Wise Convolution) replace standard *k* × *k* convolutions to extract representative features. The upper transform stage can be expressed as:


$$Y_{1}=M^{G}X_{\mathrm{up}}+M^{P1}X_{\mathrm{up}}$$

where $M^{G}$ and $M^{P1}$ are the learnable weights of GWC and PWC. $Y_{1}$ and $X_{\mathrm{up}}$ correspond to the input and output feature maps of the upper transform stage. ${Χ}_{\mathrm{low}}$ is then input to the lower transform stage, which can be expressed as:

$$Y_{2}=M^{P_{2}}X_{\mathrm{low}}\cup X_{\mathrm{low}}$$

where $M^{P_{2}}$ corresponds to the learnable weights of PWC, ∪ represents the Concat concatenation operation, and $Y_{2}$ and $X_{\mathrm{low}}$ correspond to the input and output feature maps of the lower transform stage.

After completing the transform stage, the features of the upper and lower transform stages are fused. The simplified SKNet method (19) is used to adaptively fuse the output features $Y_{1}$ and $Y_{2}$ from the transform stage. Then, global average pooling is used to collect global spatial information $S_{m}\in {\mathbb{R}}^{c\times 1\times 1}$ , which can be expressed as:


$$S_{m}=\frac{1}{H\times W}\sum_{i=1}^{H}\sum_{j=1}^{w}Y_{c}\left(i,j\right),m=1,2$$

Then, the global channel descriptors $S_{1}$ and $S_{2}$ from the upper and lower layers are stacked, and a channel soft attention mechanism is used to generate feature importance vectors $\beta_{1}$ , $\beta_{2}$ ∈ ${\mathbb{R}}^{c}$ , which can be expressed as:


$$\beta_{1}=\frac{e^{s_{1}}}{e^{s_{1}}+e^{s_{2}}},\beta_{2}=\frac{e^{s_{2}}}{e^{s_{1}}+e^{s_{2}}}$$

In the end, directed by the feature importance vectors $\beta_{1}$ and $\beta_{2}$ , the upper layer features $Y_{2}$ and lower layer features $Y_{2}$ are merged to obtain the channel reconstructed feature Y . This can be expressed as $Y=\beta_{1}Y_{1}+\beta_{2}Y_{2}$ . Thus, the two modules SRU and CRU reduce the redundancy of feature maps, leading to performance improvement while reducing computational load.

### 2.3 Multi-Dconv Head Transposed Attention module

**Figure 6:**
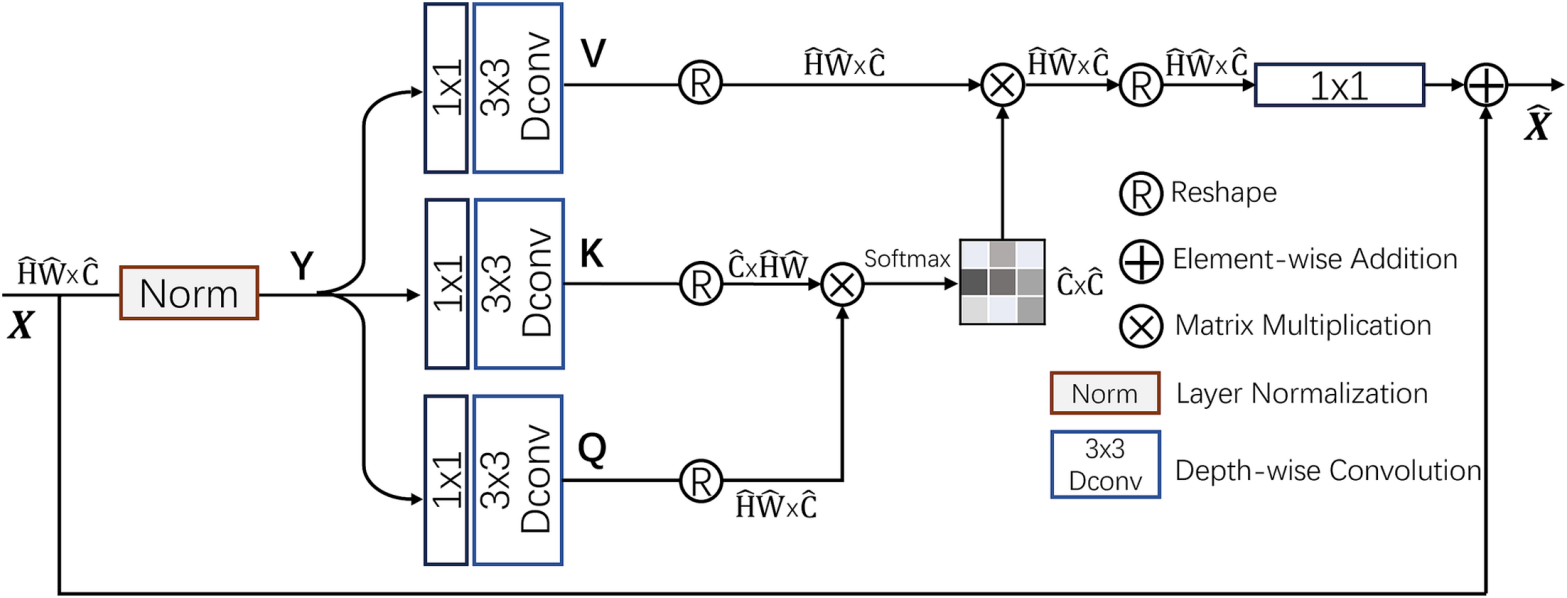
Structure of the MDTA attention module.

In traditional self-attention (SA) mechanisms (20), the dot-product interactions between keys and queries typically result in a significant increase in computational complexity as the input image size grows. In contrast, the Multi-Dconv Head Transposed Attention (MDTA) module by Zamir et al. (21) achieves better computational efficiency through innovative computational methods, as shown in Figure 6. MDTA primarily applies self-attention in the channel dimension rather than the spatial dimension by computing the cross-covariance between channels to implicitly construct a global context attention map. Additionally, by introducing point-wise convolution to enhance local context expression, MDTA further generates a global attention map through feature covariance computation.

MDTA starts from a layer-normalized tensor $Y={\mathbb{R}}^{\hat{H}\times \hat{W}\times \hat{C}}$ , generating projections for queries (Q), keys (K), and values (V). These projections first aggregate inter-channel pixel context through 1×1 convolutions and then encode intra-channel spatial context through 3 × 3 depth-wise convolutions, resulting in $Q=W_{d}^{Q}W_{p}^{Q}Y,K=W_{d}^{K}W_{p}^{K}Y\;\mathrm{and}\;V=W_{d}^{V}W_{p}^{V}Y$ . Here, $W_{p}^{\left(\text{.}\right)}$ and $W_{d}^{\left(\text{.}\right)}$ denote 1 × 1 point-wise convolutions and 3 × 3 depth-wise convolutions, respectively. By reshaping the query and key projections, their dot-product interactions generate a reverse attention map A with a size of ${\mathbb{R}}^{\hat{C}\times \hat{C}}$ instead of the traditional larger attention map. The computational process of MDTA can be summarized as:


$$\hat{X}=W_{p}\mathrm{Attention}\left(\hat{Q},\hat{K},\hat{V}\right)+X$$

$$\mathrm{Attention}\left(\hat{Q},\hat{K},\hat{V}\right)=\hat{V}\;.\;\mathrm{Softmax}\left(\frac{\hat{K}\;.\;\hat{Q}}{\alpha }\right)$$

where X and $\hat{X}$ represent the input and output feature maps; $\hat{Q}\in {\mathbb{R}}^{\hat{H}\hat{W}\times \hat{C}}$ , $\hat{K}\in {\mathbb{R}}^{\hat{C}\times \hat{H}\hat{W}}$ , and $\hat{V}\in {\mathbb{R}}^{\hat{H}\hat{W}\times \hat{C}}$ are the reshaped tensor matrices. The parameter *α* is an adjustable scaling factor that regulates the dot product magnitude between *K* and *Q* prior to applying the Softmax function. Like conventional multi-head self-attention, the channels are split into several “heads” to simultaneously learn distinct attention maps.

By integrating the MDTA module into image classification models, the ability of the model to capture image features is significantly improved. This enhanced self-attention mechanism optimizes inter-channel interactions, effectively increasing the model’s sensitivity to key visual information, thereby improving classification accuracy.

## 3 Experimental results and discussion

This section primarily delves into assessing how effectively the proposed model identifies and diagnoses aortic stenosis along with determining its severity. Section 3.1 introduces the publicly available dataset used for the experiment, Section 3.2 explains the experimental parameter settings, and Section 3.3 presents the analysis and discussion of the model’s performance based on different evaluation metrics.

### 3.1 Experimental dataset

This study uses the publicly available dataset from Tufts University (Tufts Medical Echocardiogram Dataset, TMED) (22) to evaluate and test the proposed model. It is worth noting that the dataset currently has two versions: TMED-1 and TMED-2. In this study, TMED-2 is used for both diagnosis and severity classification, while TMED-1 is employed to further validate the model’s diagnostic performance for AS. The TMED-1 dataset contains data from 260 patients, with each patient’s images labeled for AS diagnosis (none, mild/moderate, severe) and image view types (PLAX, PSAX, others). Compared to TMED-1, TMED-2 includes additional views such as A2C and A4C, provides more detailed severity labels, and contains higher-resolution images. Additionally, the images in TMED are all sized at 64 × 64. The TMED-2 dataset contains 599 studies with 17,270 fully annotated images, including different views (PSAX, PLAX, A2C, A4C) and severity labels (none, mild, mild to moderate, moderate, severe). All images have been preprocessed, leveraging metadata from the original DICOM files to ensure inclusion of only 2D TTE images from each study, while excluding Doppler images, M-mode images, and color flow images. The images were resized to 112 × 112 and saved in PNG format.

For the binary classification diagnosis of aortic stenosis, we grouped images labeled “Mild,” “Mild to Moderate,” “Moderate,” and “Severe” as “AS,” and “None” as “no_AS.” For severity recognition, images labeled “Mild,” “Mild to Moderate,” and “Moderate” were grouped as “MildtoMod_AS,” and “Severe” was grouped as “Severe_AS,” removing the “no_AS” label. Lastly, data augmentation was applied to the images, including rotation, flipping, brightness adjustment, and scaling. The TMED-2 dataset was then partitioned into training, validation, and test sets in a ratio of 6:2:2, as illustrated in Table 1. In addition, to reduce the impact of randomness on the performance of RSMAS-Net and to ensure the reliability of the results, the dataset splitting process was repeated five times.

**Table 1:** The division of the augmented experimental dataset, including the number of samples in the training, validation, test sets, and the total number of sample.

	AS diagnosis			AS severity		
Split	no_AS	AS	Total	MildtoMod	Severe	Total
Train	5,790	45,530	51,320	24,095	22,665	46,750
Validation	1,835	16,175	18,010	8,035	7,555	15,590
Test	1,775	16,235	18,010	8,030	7,560	15,590

### 3.2 Experimental setup and evaluation metrics

Experimental hardware configuration: AMD R7 5800X3D CPU, Nvidia RTX 4090 GPU, 32GB RAM; Software configuration: Windows 11 OS, Pytorch version:1.12.1, Python version:3.10.0, CUDA version:12.3 architecture. Parameter settings: The epochs are configured to 100, and the batch size is configured to 32, As the optimizer, AdamW (23) is used with an initial learning rate of 1 × 10^−4^, using the cross-entropy loss function, using pre-trained parameters from the ImageNet dataset (24). A default threshold of 0.5 is used to convert predicted probabilities into binary labels when calculating accuracy, precision, recall, and *F*_1_-score.

The evaluation metrics and tools for model performance include:

1.
$\mathrm{Accuracy}=\frac{TP+TN}{TP+TN+FP+FN}$

2.
$\mathrm{Precision}=\frac{TP}{TP+FP}$

3.
$\mathrm{Recall}=\frac{TP}{TP+FN}$

4.
$F_{1}-\mathrm{score}=2\times \frac{\mathrm{Precision}\times \mathrm{Recall}}{\mathrm{Precision}+\mathrm{Recall}}$

5. ROC curve, where an AUC value closer to 1 indicates better classification performance.
6. PR (precision-recall) curve, where the area under the curve (AP, average precision) is larger, indicates better model performance.
7. Confusion matrix: a matrix that compares the model’s predictions with the actual labels.
8. Model parameters: the overall count of trainable parameters within the model. Fewer parameters usually mean higher computational efficiency.
9. Prediction time: the time it takes for the model to handle a single sample or a batch of samples.

### 3.3 Results analysis and discussion

To validate the effectiveness and performance of RSMAS-Net in identifying AS and its severity, this study sets up a comparative experiment involving multiple models and metrics for AS diagnosis. Currently, there is limited research on binary AS identification using the TMED-2 dataset. Therefore, inspired by previous research (which conducted AS classification on the TMED-1 dataset), this paper selects ResNet50, EfficientNetV2 (25), VGG16 (26), SqueezeNet (27), and MobileNet (28) as comparison models. To better compare the models, all comparison models were trained and validated according to the experimental parameter settings in Section 3.2. Additionally, an AS severity classification experiment was set up to evaluate the model’s ability to distinguish between “Mild to Moderate” and “Severe” AS.

#### 3.3.1 Model performance in AS diagnosis binary classification and comparative analysis

**Figure 7:**
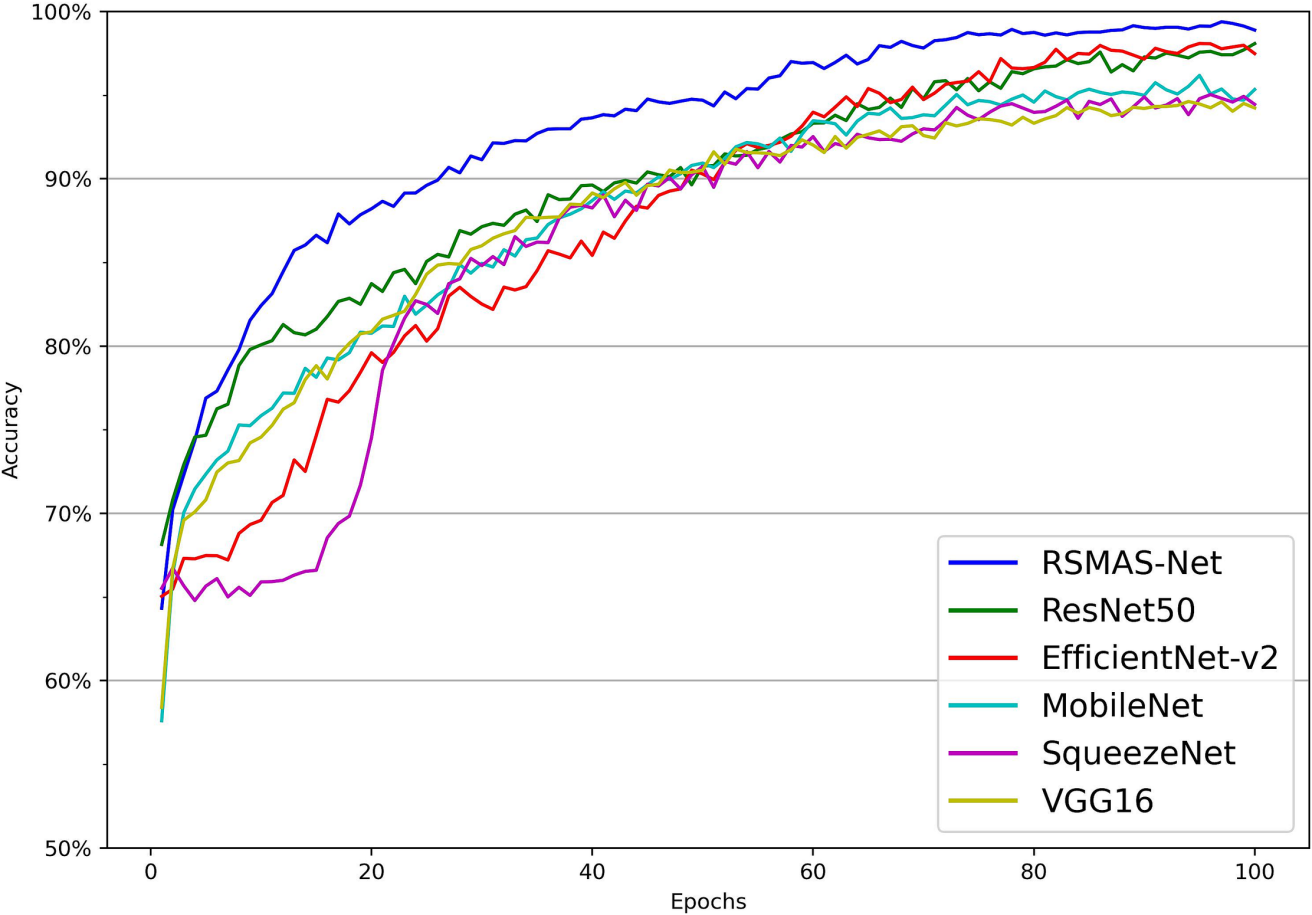
Comparison of training set accuracy across multiple models.

**Figure 8:**
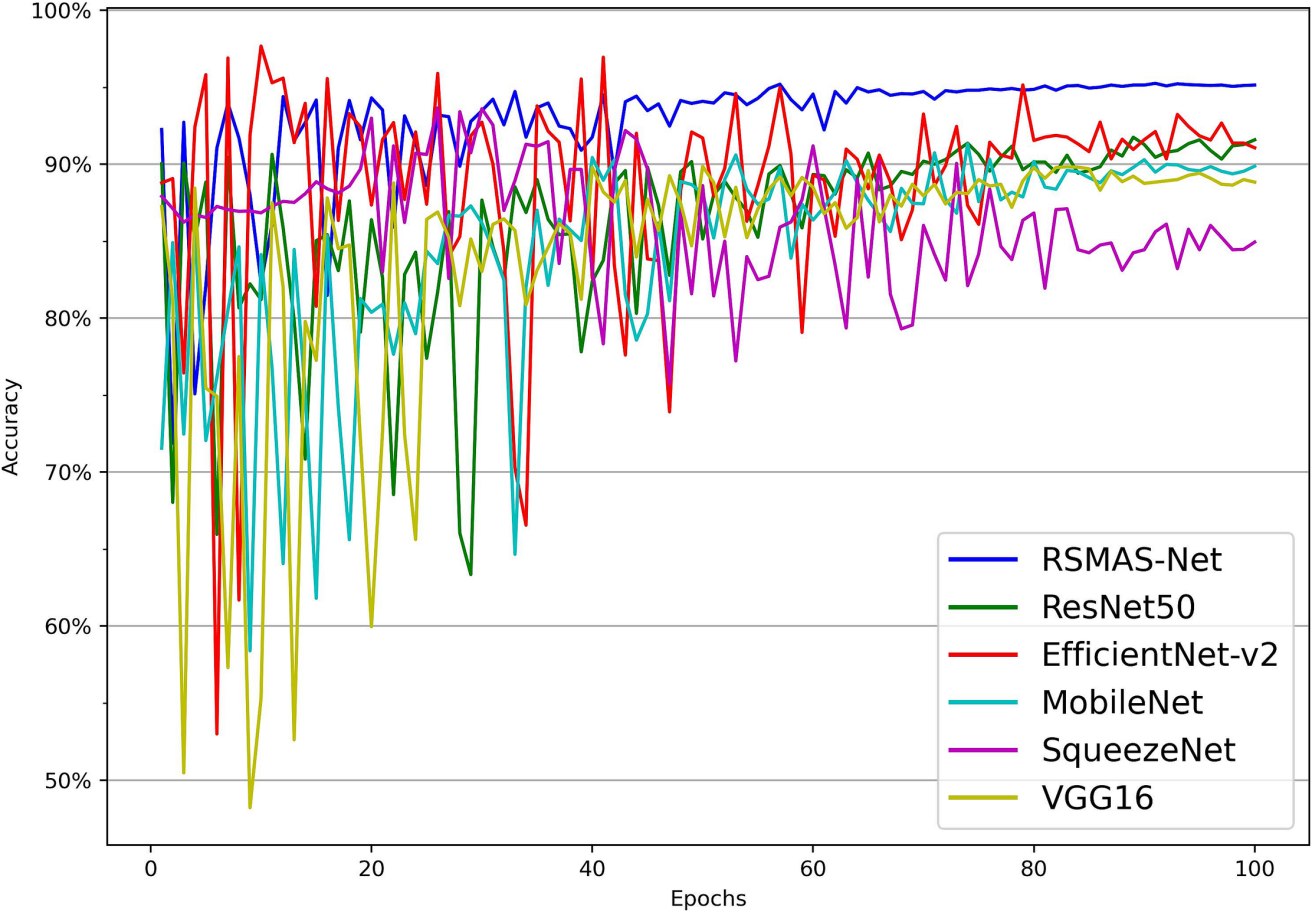
Comparison of validation set accuracy across multiple models.

As shown in Figures 7, 8, the accuracy of multiple models on the training and validation sets is presented. All models demonstrate good performance in AS identification and diagnosis. In terms of training accuracy, the proposed model converges quickly, achieving high accuracy early on, and then grows steadily. The curve is higher and smoother compared to other models, indicating high efficiency and strong generalization ability during the learning process. In contrast, the accuracy of the comparison models grows slowly in the early stages of training and does not reach the same level as even in the later stages of training. In terms of validation accuracy, all models exhibit significant fluctuations in the early stages, but RSMAS-Net shows more stability and consistency in the middle and later stages, maintaining an accuracy of over 90%, higher than other models.

**Figure 9:**
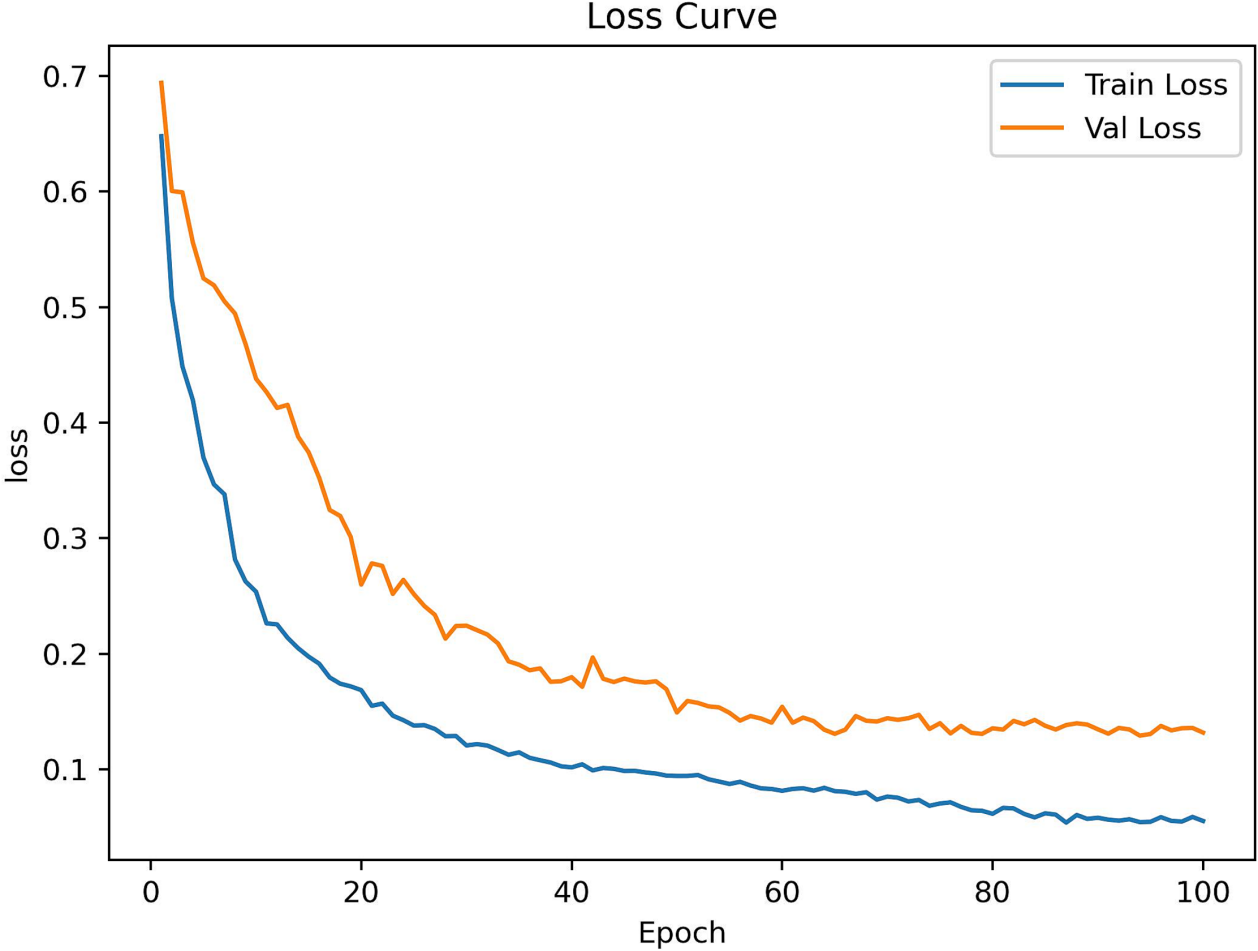
RSMAS-Net training and validation loss curves.

Additionally, Figure 9 shows RSMAS-Net’s performance on the loss curves. Both training loss and validation loss rapidly decrease from the initial value, and the loss curves quickly converge. This indicates that the model is effectively learning image features and continuously optimizing classification performance. The training loss is slightly lower than the validation loss, and both tend to stabilize as the epochs increase, without significant overfitting. The loss curves demonstrate good convergence and stability of the model during training and validation.

Tables 2, 3 present more results. In Table 2, all models have good metric parameters, RSMAS-Net achieves the highest accuracy, precision, recall, and *F*_1_-score, reaching 94.67% ± 0.32, 91.93% ± 0.48, 96.95% ± 0.35, and 94.37% ± 0.30%, respectively, which represent the average results over five independent runs. Notably, when contrasted with ResNet50, our improved model with SCConv and MDTA attention modules as the backbone enhances accuracy by 2.44%. Additionally, the inclusion of SCConv and MDTA attention modules reduces redundant features and improves computational efficiency. As shown in Table 3, compared to non-lightweight design models, our model has fewer parameters and faster prediction times. Overall, thanks to the SCConv and MDTA attention modules, the proposed model achieves the best metrics in comparative experiments on the training and validation sets. The accuracy has increased while the number of parameters has decreased, demonstrating that the proposed model performs exceptionally well in identifying and classifying AS.

**Table 2:** Performance metrics of multiple models on the AS diagnostic classification task, with results obtained on the test split.

Model	Accuracy	Precision	Recall	*F*_1_-score
SqueezeNet	88.69%	86.92%	90.21%	88.53%
VGG16	88.73%	86.95%	90.53%	88.70%
MobileNet	88.94%	87.96%	90.65%	89.28%
EfficientNetV2	91.98%	89.97%	94.12%	92.00%
ResNet50	92.23%	90.06%	94.78%	92.36%
RSMAS-Net	94.67%	91.93%	96.95%	94.37%

**Table 3:** Comparison of parameters and prediction time between the RSMAS-NET and non-lightweight models.

Model	Parameters (millions)	Prediction time (ms)
ResNet-50	25.63	0.59
EfficientNetV2-S	21	0.66
VGG16	138	0.68
RSMAS-Net	17.6	0.50

**Figure 10:**
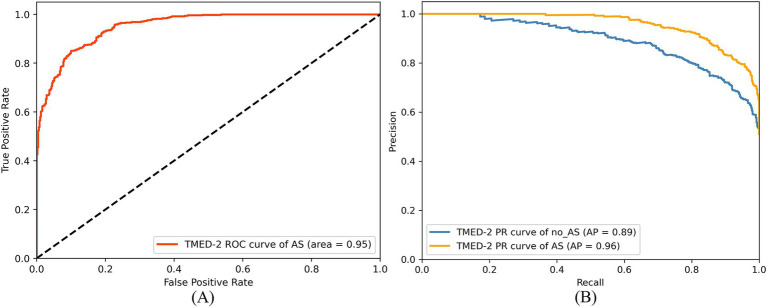
ROC curve **(A)** and PR curve **(B)** of RSMAS-Net on the AS diagnostic classification.

Additionally, the study utilized a ROC curve to evaluate the model’s performance in distinguishing between the two classes. As shown in Figure 10A, the AUC for the AS class was 0.95, indicating strong classification performance in identifying the AS class. This high AUC value demonstrates that the RSMAS-Net model effectively distinguishes AS from no_AS cases, which is crucial for accurate diagnosis of AS. In cases of class imbalance, the Precision-Recall (PR) curve serves as an essential metric for classification performance evaluation. As shown in Figure 10B, the average precision (AP) for the AS class was 0.96, while the AP for the no_AS class was 0.89. Although a standard binary classification task typically yields a single PR curve by treating one class as positive, we intentionally plotted two separate PR curves by alternately setting “AS” and “no_AS” as the positive class. This approach was adopted to provide a more comprehensive evaluation of the model’s ability to correctly identify both categories. In clinical practice, accurately recognizing non-stenotic (no_AS) cases is equally important to avoid misdiagnosis or overtreatment. Therefore, this dual-curve analysis highlights that the proposed model not only excels in detecting AS but also performs well in ruling out AS, which enhances its reliability in real-world applications.

Since the dataset contains more AS samples than no_AS samples, the model tends to learn AS-class features more effectively, leading to slightly better recognition performance for AS compared to no_AS. However, this performance gap has been minimized through data augmentation. Overall, the proposed RSMAS-Net model demonstrates high accuracy in distinguishing between AS and no_AS cases, confirming its effectiveness in the binary classification task for AS diagnosis.

The strong performance of RSMAS-Net demonstrates its potential clinical value in the diagnosis of AS. The model effectively extracts subtle and critical structural features from static echocardiographic images, improving recognition accuracy in early-stage or borderline cases, which are often difficult to assess in clinical practice. With fewer parameters and high computational efficiency, it is suitable for deployment in real-time settings such as bedside examinations or portable devices. Additionally, RSMAS-Net maintains stable performance in both AS and no_AS classification, even under class imbalance, helping reduce misdiagnosis and unnecessary interventions while enhancing the overall reliability and generalizability of AS screening.

#### 3.3.2 Further validation of AS diagnosis performance

**Figure 11:**
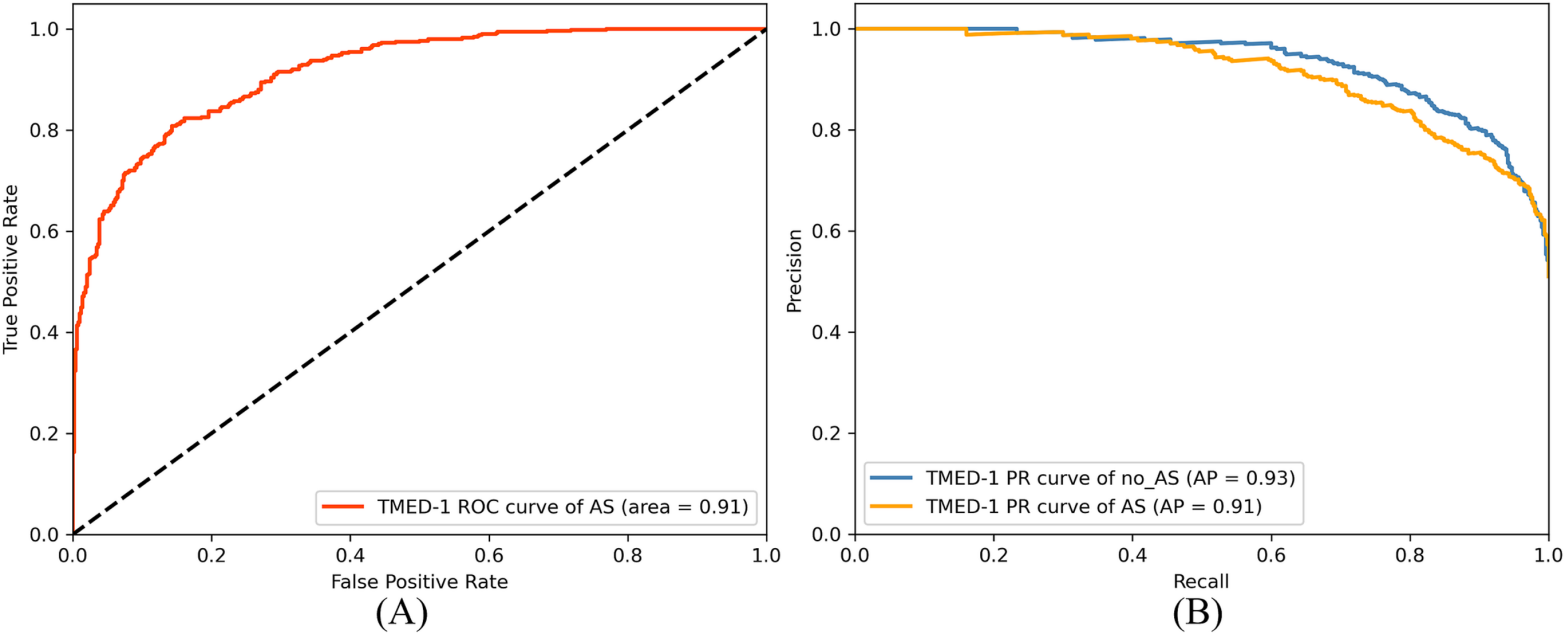
ROC curve **(A)** and PR curve **(B)** of RSMAS-Net on AS diagnostic classification in TMED-1 validation.

The study further validated the AS classification performance of the model on the TMED-1 dataset. As shown in Figures 11A,B, the ROC curve indicates that the AUC of AS was 0.91. In the PR curve results, the AP for the no_AS class was 0.93, while the AP for the AS class was 0.91, demonstrating that the model maintains high precision even at high recall rates.

**Figure 12:**
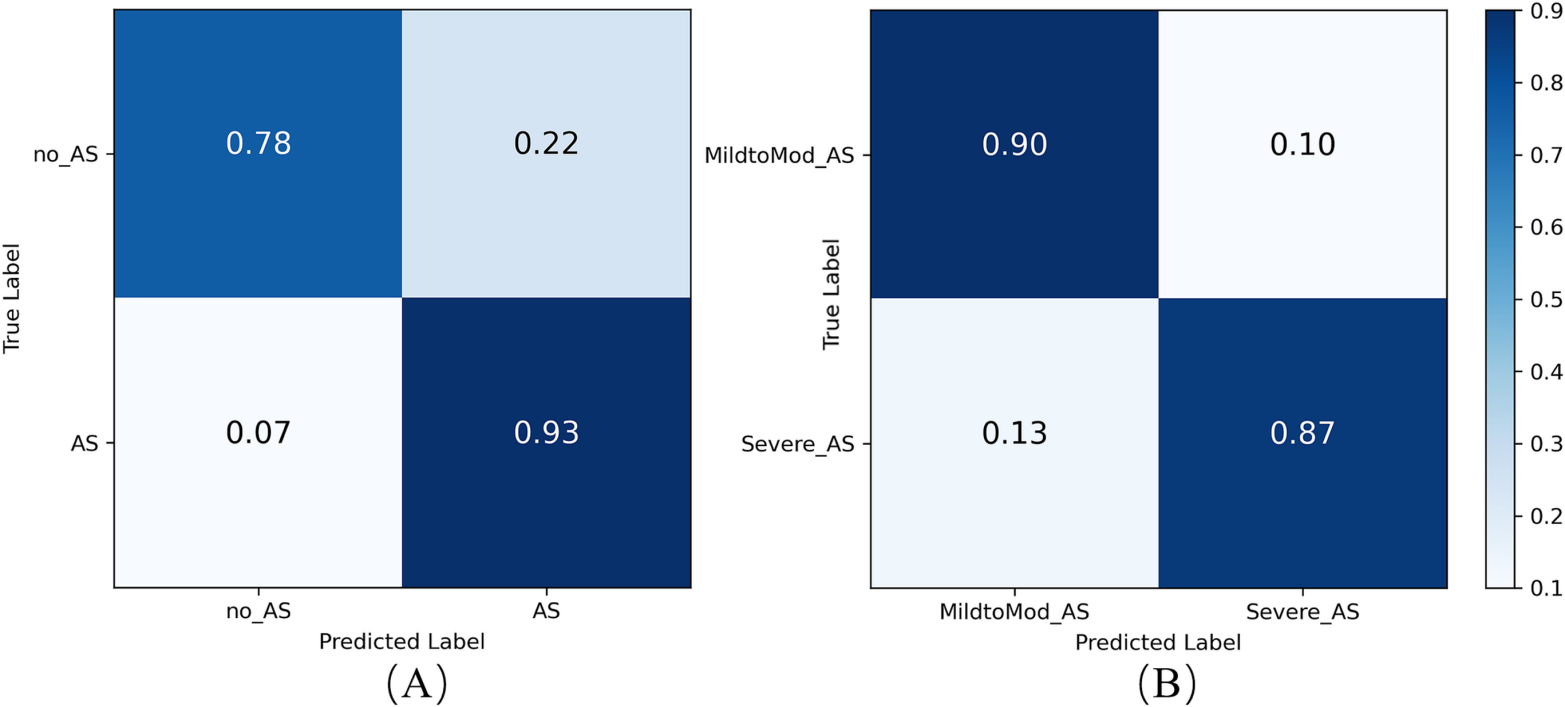
Confusion matrix of the RSMAS-Net on the AS **(A)** diagnostic and **(B)** severity classification.

The confusion matrix analysis, presented in Figure 12A, shows that the model achieved a prediction accuracy of 78% for the no_AS class and 93% for the AS class. These results indicate that the model can still achieve accurate and sensitive diagnosis of AS even when applied to external data. This demonstrates the model’s strong generalization ability beyond the training dataset. External validation is a critical step in the clinical translation of AI-based medical models, as it helps verify their performance across diverse data sources and patient populations. The stable performance under class imbalance further highlights the robustness and reliability of the model for practical AS screening in real-world settings.

#### 3.3.3 Model performance analysis for AS severity classification

**Figure 13:**
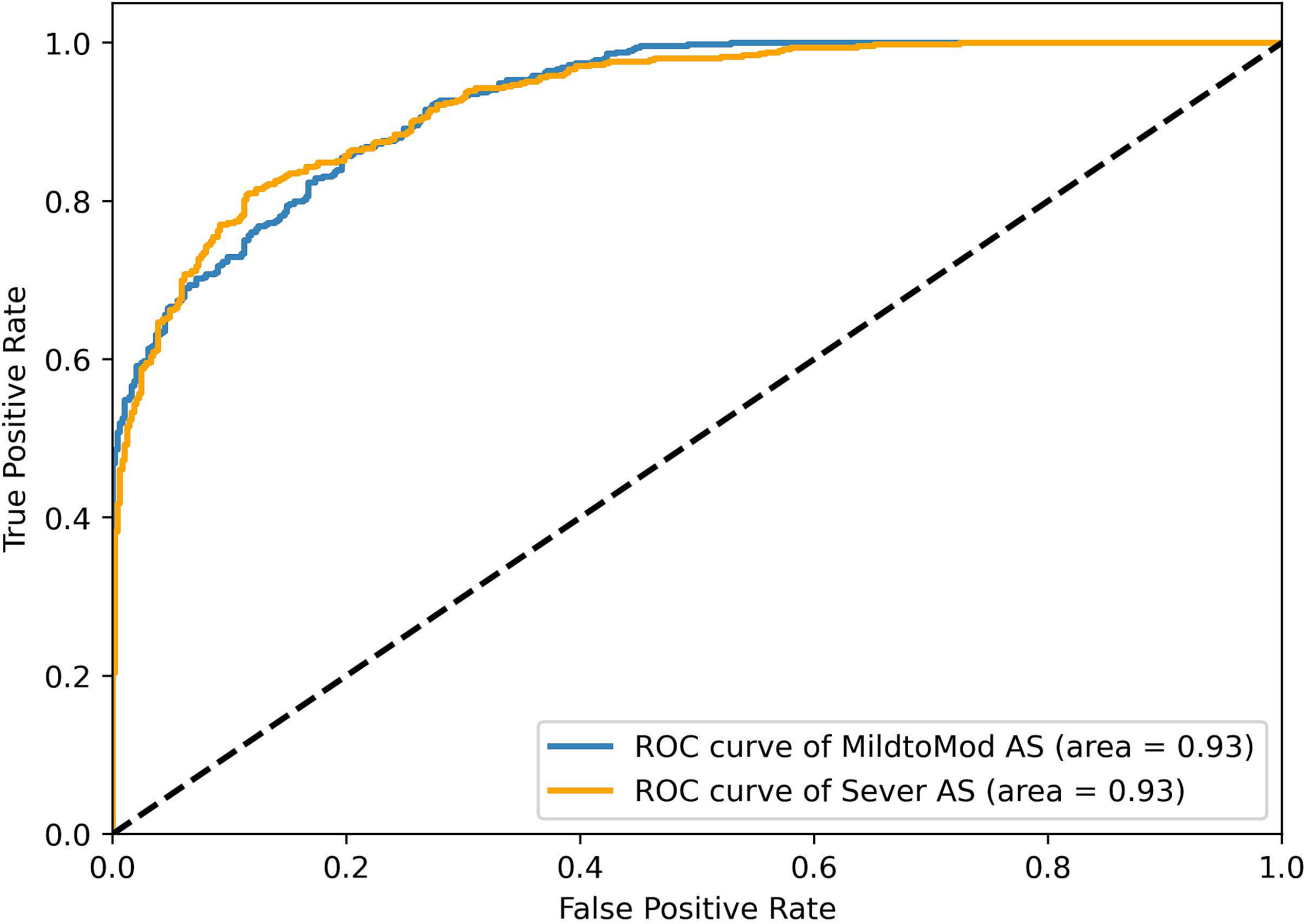
ROC curve results of the RSMAS-Net on the AS severity classification.

To assess RSMAS-Net effectiveness in classifying the severity of AS, we used ROC curves and confusion matrices for result analysis. Figure 13 shows the ROC curves, with the blue and orange curves representing the ROC curves for “MildtoMod_AS” and “Severe_AS,” respectively. Both curves have an AUC value of 0.93, suggesting that the model possesses a high discriminative capacity. The high AUC values for both categories indicate that the model performs excellently in correctly classifying mild to moderate and severe AS instances. Figure 12B shows the confusion matrix for the binary classification task of AS severity. True positives (mild to moderate correctly classified as mild to moderate), false positives (mild to moderate misclassified as severe), true negatives (severe correctly classified as severe), and false negatives (severe misclassified as mild to moderate). The matrix data shows that the model has a high classification accuracy for both categories, with 90% of mild to moderate AS samples and 87% of severe AS samples being correctly classified, resulting in a low overall classification error rate. Combining the ROC curve and confusion matrix evaluation metrics, the proposed model has demonstrated robust performance and accuracy in identifying AS severity and diagnosing AS.

These results demonstrate that RSMAS-Net is capable not only of identifying AS but also of accurately classifying its severity, which is essential for risk stratification and treatment planning in clinical practice. Severity grading of aortic stenosis plays a critical role in determining clinical management strategies, such as the timing of surgical intervention or monitoring frequency. The high AUC values and classification accuracy for both mild-to-moderate and severe AS indicate that the model can effectively distinguish between different disease stages. This supports its potential role in assisting clinicians with more precise diagnosis and timely intervention.

## 4 Conclusion

In this study, we propose an improved deep learning model, RSMAS-Net, based on ResNet50, integrating SCConv and MDTA attention modules to accurately identify the presence of AS in multi-view echocardiography. Through extensive training and validation on the TMED-2 dataset, our model outperformed several popular deep learning models in AS classification across key performance metrics, including accuracy and *F*_1_-score. Notably, compared to the original ResNet50, our model achieved higher classification accuracy while reducing the number of parameters, demonstrating superior model efficiency. To further evaluate the generalization capability, we conducted additional testing on the TMED-1 dataset, where our model also achieved good classification performance. This result suggests that the proposed method is not only effective on TMED-2 but can also maintain strong robustness across different data distributions. Additionally, in the AS severity classification task, our model effectively distinguished mild-to-moderate AS from severe AS, highlighting its clinical value in echocardiography-based disease grading. More importantly, our model demonstrates high accuracy, efficiency, and fast inference speed, enabling precise AS identification and assessment to assist clinicians in real-time computer-aided diagnosis (CAD). This advancement supports early intervention and treatment, improving patient outcomes.

While this study first introduces SCConv and MDTA modules into cardiac ultrasound analysis and verifies their effectiveness in handling complex and dynamic cardiac structures, several areas require further optimization:

1. Enhancing performance on more complex or ambiguous ultrasound images: Our model performs well on high-quality echocardiographic images, but its classification accuracy decreases when dealing with artifacts, low signal-to-noise ratio (SNR), or variations in probe angles. This suggests that the model may be sensitive to data quality. Future research could incorporate adversarial training or image enhancement strategies to improve model robustness in challenging imaging scenarios. In addition, optimizing the decision threshold used to convert predicted probabilities into class labels may help balance sensitivity and specificity more effectively. Rather than relying on a fixed threshold, future work could explore adaptive thresholding strategies based on validation performance, ROC analysis, or specific clinical requirements. Such optimization may be particularly beneficial in cases involving uncertain image quality or class imbalance, where threshold tuning can significantly impact diagnostic accuracy.
2. Improving adaptability to diverse patient populations and extreme cases: Although the model has demonstrated good generalizability on TMED-1 and TMED-2, it has primarily been trained on a specific echocardiography dataset; to enhance cross-population adaptability, future studies should validate the model on more diverse datasets, including patients of different ethnicities, age groups, and medical histories; Approaches such as transfer learning and data augmentation could be explored to expand the model’s applicability across broader patient populations.
3. Enhancing model interpretability for clinical applications: Although SCConv and MDTA improve feature extraction, clinicians require intuitive explanations for the model’s decision-making process; Future research could integrate Explainable AI (XAI) techniques, such as Grad-CAM or attention-based visualization methods, to provide more interpretable decision rationales, improving clinical usability and trustworthiness.
4. Further optimizing computational efficiency for real-time clinical applications: While our model already reduces parameter complexity compared to ResNet50, computational cost remains a concern in resource-constrained environments (e.g., portable ultrasound devices or edge computing platforms); Future optimizations may explore lightweight architectures (e.g., MobileNet, EfficientNet) or implement model quantization and pruning techniques to reduce inference time and enhance real-world deployability.

Overall, this study not only proposes an efficient and accurate echocardiographic analysis model but also pioneers the application of SCConv and MDTA modules in cardiac imaging. Future research will focus on model optimization, dataset diversification, and explainability enhancement to further improve the model’s clinical adaptability and scalability. We hope that the findings of this study will advance the automation of echocardiographic disease diagnosis, providing clinicians with more precise and efficient decision-support tools and ultimately driving the integration of AI into the medical and healthcare domain.

## Data Availability

The original contributions presented in the study are included in the article/supplementary material, further inquiries can be directed to the corresponding authors.
